# Targeted Gene Candidates for Treatment and Early Diagnosis of Age-Related Macular Degeneration

**DOI:** 10.1155/2021/6620900

**Published:** 2021-02-02

**Authors:** Emine Cinici, Ozge Caglar, Mehmet Enes Arslan, Nilay Dilekmen, Bahadır Utlu, Adil Mardinoglu, Hasan Turkez

**Affiliations:** ^1^Department of Ophthalmology, Faculty of Medicine, Atatürk University, Erzurum, Turkey; ^2^Department of Molecular Biology and Genetics, Faculty of Science, Erzurum Technical University, Erzurum, Turkey; ^3^Department of Ophthalmology, Palandöken State Hospital, 25100 Erzurum, Turkey; ^4^Ophthalmology, Regional Training and Research Hospital, Health Sciences University, Erzurum, Turkey; ^5^Science for Life Laboratory, KTH-Royal Institute of Technology, SE-17121 Stockholm, Sweden; ^6^Centre for Host-Microbiome Interactions, Faculty of Dentistry, Oral & Craniofacial Sciences, King's College London, London SE1 9RT, UK; ^7^Department of Medical Biology, Faculty of Medicine, Atatürk University, Erzurum, Turkey

## Abstract

Age-related macular degeneration (AMD) is an eye disease that impairs the sharp and central vision need for daily activities. Recent advances in molecular biology research not only lead to a better understanding of the genetics and pathophysiology of AMD but also to the development of applications based on targeted gene expressions to treat the disease. Clarification of molecular pathways that causing to development and progression in dry and wet types of AMD needs comprehensive and comparative investigations in particular precious biopsies involving peripheral blood samples from the patients. Therefore, in this investigation, dry and wet types of AMD patients and healthy individuals were aimed at investigating in regard to targeted gene candidates by using gene expression analysis for the first time. 13 most potent candidate genes involved in neurodegeneration were selected via in silico approach and investigated through gene expression analysis to suggest new targets for disease therapy. For the analyses, 30 individuals (10 dry and 10 wet types AMD patients and 10 healthy people) were involved in the study. SYBR-Green based Real-Time PCR analysis was performed on isolated peripheral blood mononuclear cells (PBMCs) to analyze differentially expressed genes related to these cases. According to the investigations, only the *CRP* gene was found to be upregulated for both dry and wet disease types. When the downregulated genes were analyzed, it was found that 11 genes were commonly decreased for both dry and wet types in the aspect of expression pattern. From these genes, *CFH*, *CX3CR1*, *FLT1*, and *TIMP3* were found to have the most downregulated gene expression properties for both diseases. From these results, it might be concluded that these common upregulated and downregulated genes could be used as targets for early diagnosis and treatment for AMD.

## 1. Introduction

Age-related macular degeneration (AMD) is a clinical condition that affects individuals aged 50 years and over and causes a progressive decrease in visual acuity by progressing with pigmentary and atrophic changes in the macula [1]. A neurodegenerative disease affecting the photoreceptor, retinal pigment epithelium (RPE), Bruch's membrane, and choriocapillaris in the macula is considered as the AMD disease. It is the most common cause of vision loss in people aged 65 and over in developed countries. Its frequency is 10% between the ages of 65 and 75 and 25% over the age of 75 [2]. It is estimated that the number of patients will be 288 million by 2040 [3]. Classically, there are two subgroups of AMD: (I) atrophic form (dry type) and (II) exudative form (wet type). Dry-type AMD is nonneovascular and typically leads to progressive degeneration of RPE and photoreceptors, resulting in chorioretinal and geographic atrophy. The exudative neovascular form is the wet type that results in central vision loss due to choroidal neovascularization directed to the subretinal macular region and the fastest progressing form of AMD. The accumulation of membranous wastes between the RPE and the Bruch's membrane is called drusen. Although drusen was previously defined only as lipofuscin accumulation, microscopic examinations performed with different immunohistochemistry dyes also found that it contains lipids, carbohydrates, and proteins such as amyloid, fibronectin, vitronectin, and complement factors [4].

There are genetic and environmental risk factors in the etiopathogenesis of AMD. The impacts of genetic and environmental factors in the development of AMD are 71% and 29%, respectively. Several genes have been thought to affect genetic risk [5]. In recent years, mutations or polymorphisms that can affect the risk of developing AMD have also been investigated. Therefore, research on the genetic component of AMD has been the focus of attention in the last 10 years. Although it is known that the disease occurs with the effect of multiple genetic factors, it is important to determine the genetic components so that the pathophysiology of the disease is understood in the light of the knowledge of these components and the connections between different diseases [6].

Genetic studies have shown that some genes may be associated with AMD. The number of studies aimed at determining the genomic regions affected during the pathogenesis of AMD is remarkable. The Retina International Database showed 16 genes associated with AMD. In this sense, the *CFH* gene is the first important gene that is associated with AMD. Complement factor B, C2, and C3 can also be listed among other important genes [7]. The experiments on pluripotent stem cells propounded *AMPK*, *IGF1*, *MTOR*, *PPARGC1A*, and *SIRT1* genes were related to both aging stress response and AMD pathology [8]. Extensive researches pointed out that more genes might be associated with AMD. *ABCA1* [9], *ARMS2* [10], *APOE* [11, 12], *CFH* [13], *CX3CR1* [14], *CCR5* [15], *ELOVL2* [16], *HTRA1* [17], *KCTD10* [18], *TIMP3* [19], and *VEGFA* [20] are just a few of these genes reported being related to AMD. Besides, the methylation levels of certain genes including *CDKN1C*, *EZR*, *IGF2*, *NOP56*, and *SLC2A1* were altered in blood tissues of patients with AMD [21]. Currently, there is some treatment options such as anti-VEGF for Wet-AMD but not available for Dry-AMD.

Elucidation of underlying molecular mechanisms that giving rise to development and progression in both types of AMD needs comprehensive and comparative investigations in particular precious biopsies such as peripheral blood, cerebrospinal fluid, RPE/choroid, and induced pluripotent stem cells-derived retinal pigment epithelium (iPSC-RPE) from the patients. Moreover, blood plasma or blood cells as well as retinal tissues of the patients are considered as favorable for deeply evaluation of differentially expressed molecular and genetic factors including genes, RNA types, and inflammatory elements and their association with AMD [22, 23]. In this context, molecular genetic assessments particularly expression profiling of associated or targeted genes are very crucial to explore novel curation or diagnosis strategies for both types of AMD. Further, investigating various genes and their relationship with the different types of this disease could serve in constituting target specific therapies. Therefore, in this study, dry and wet types of AMD patients and healthy individuals were aimed at investigating in regard to targeted gene candidates by using gene expression analysis for the first time. For this aim, 13 most potent candidate genes that associated with neurodegeneration were chosen via in silico approaches for analyzing gene-disease relationship to propose molecular diagnosis points and gene-targeted treatments against two types of AMD.

## 2. Methods

### 2.1. Patients

A total of 20 female patients with Dry-AMD (*n* = 10, 57.9 ± 6.1) and Wet-AMD (*n* = 10; 59.4 ± 7.7) who routinely applied to the eye disease clinic, participated in this study. Those with systemic diseases (diabetes mellitus, etc.) or ophthalmic diseases that may cause pathology in the retina were excluded from the study. As the control group, 10 nonsmoker females who came to the internal medicine clinic for routine control were included in the same age range (60.8 ± 6.9). Socio-demographic and clinical details were recorded by submitting the standard questionnaire to the participants at the time of recruitment. The current study was carried out with the approval of Ethics Committee of Atatürk University, Faculty of Medicine, with letter number 51 and dated 22.04.2009.

### 2.2. Isolation of Peripheral Blood Mononuclear Cells (PBMCs)

Blood samples were taken from 20 patients who applied to Atatürk University Ophthalmology Outpatient Clinic in the year of 2020. For PBMC isolation, 10 ml of blood was taken from the participants by using EDTA blood collection tubes. PBMC isolation was carried out via the Ficoll density method. Briefly, whole blood was added into the vial including PBS in a ratio of 1 : 1. Then, the blood diluted with PBS is slowly dropped into the tube with Ficoll. The tube was centrifuged at 18-24°C, 400 g for 30 minutes. After centrifugation, the PBMC layer was transferred to a new 50 ml vial so that the layers do not deteriorate. The PBMC fraction was washed by adding approximately 3 ml of PBS. The tube was centrifuged at 18-24°C for 10 min at 100 g. The supernatant was removed, and the pellet was washed again.

### 2.3. RNA Isolation and cDNA Synthesis

RNAs were extracted from PBMC using PureLink™ RNA Mini Kit (Invitrogene®, USA) procedure. To evaluated RNA purity and concentrations, a UV-visible spectrophotometer (NanoDrop®, USA) and bioanalyzer (Agilent Technologies, USA) was used. The samples were stored at -20°C until the next run. cDNA was synthesized from total RNA (QuantiTect Reverse Transcription Kit, Qiagen).

### 2.4. Real-Time PCR

The selected candidate gene expression profiles were investigated for two different disease groups by the Real-time PCR technique. cDNAs for Real-time PCR analysis were synthesized by using 100 ng RNA sample according to the manufacturer's instructions (QuantiTect SYBR® Green PCR Kits). Primer pairs used for all genes are given in Table 1. Real-time PCR was performed via the use of the SYBR-Green method on a Biorad iCycler iQ5 detection system [24].

### 2.5. Statistical Analysis

Statistical analysis of data obtained from experiments was performed via the use of the GraphPad Prism® version 7.0 software. One-way ANOVA and Tukey analysis were used for comparison evaluations, and the criterion for statistical significance was *p* < 0.05.

## 3. Results

**Figure 1:**
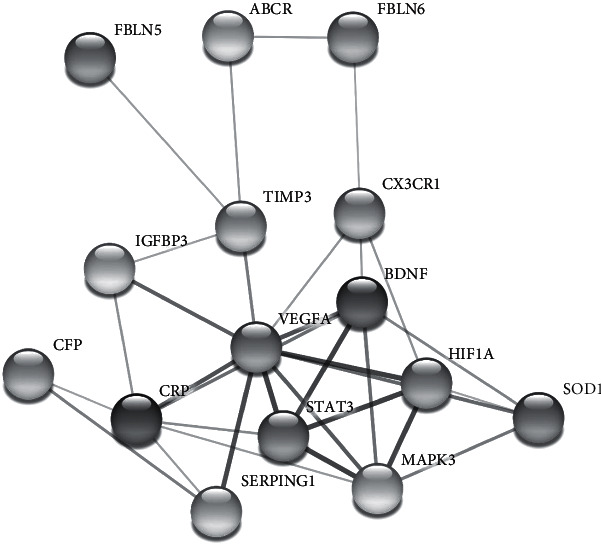
Protein-protein interaction network analysis (STRING v10) of differentially expressed genes involved in neurodegeneration.

PBMCs were isolated from blood collected from donors with dry and wet AMD diseases and no symptoms. Real-time PCR analysis was performed in triplicate for each sample in order to investigate the expression profiles of the 13 determined genes which are related to neurodegeneration via bioinformatic analysis. Gene-disease relationship was investigated by using STRING v10 multiple protein comparison analysis [25]. Candidate 13 genes were compared each other and also 3 genes that was known to have direct relationship with AMD types. These genes were correlated with the previous reports recorded in literature on the fibulin-5 (*FBLN5*) [26], fibulin-6 (*FBLN6*) [27], and photoreceptor cell–specific ATP-binding cassette transporter (*ABCR*) genes [28]. 13 candidate genes and relationship with three known proteins are schematized in Figure 1. The fold changes (F.C.) of the expression profiles for the diseases with real-time PCR analysis are presented in Table 2. According to the analysis, only the *CRP* gene expression was found to be increased in each disease group. Also, it was found that the *CX3CR1*, *FLT1*, *IGFBP3*, *MAPK3*, *SOD1*, *STAT3*, *VGEFA*, *TIMP3*, and *SERPING1* gene expressions were decreased in both Dry-AMD and Wet-AMD patients. On the other hand, the *BDNF* gene expression was observed to be upregulated for Dry-AMD but downregulated for Wet-AMD. Moreover, *FLT1* and *TIMP3* genes were investigated as the highest downregulated common genes for Dry-AMD and Wet-AMD (over a 5-fold change in gene expressions).

## 4. Discussion

The genetic variants of complex diseases are difficult to understand because they involve the interactions of many factors at the same time and complicated states of other diseases [29, 30]. However, investigation of the mechanisms of human diseases has offered new perspectives for the diagnosis and treatment of diseases. Identifying new genetic factors that are effective in the pathogenesis of the disease also has pointed new ways to prevent the disease. Genetic factors have known to play an important role in the development of AMD [31]. Indeed, it was reported that the 57% of the genes responsible for the AMD risk in RPE, choroidal, and neural retinal cells were covered the most 25% of expressed genes, and 9% these genes were also covered the most 1% of expressed genes [32]. Alike, prominent differences in expression profiles of the genes were detected in RPE as compared to iris pigment epithelium [33]. Hence, gene expression studies may execute the hidden correlations between gene expression and genetic variation and conduce to introduction of targeted gene candidates that result in AMD pathologies [34]. Additionally, neurotrophic factors that regulate the proliferation, differentiation, and functioning of neurons represent a class of regulatory proteins of nerve tissue [35].

BDNF, brain-derived neurotrophic factor, is one of the neurotrophic factors involved in the survival and differentiation of retinal ganglion cells (RGCs) and axon and dendrite development in RGCs. In many studies, the expression of BDNF and its receptors have been shown in different eye structures such as outer and inner retinal structures [36]. Nonetheless, the role of expression profile by the gene, BDNF, on AMD is still unclear and needs to be further elucidated. In a previous study, serum BDNF levels were evaluated via specific enzyme-linked immunosorbent assay (ELISA), and it was reported that serum BDNF levels were significantly higher in AMD patients in comparison to healthy subjects. Based on the involvement of retina in nervous system, these observed alterations in BDNF levels would associated with the process of retinal degeneration in AMD [37]. In supporting this finding, our real-time results showed that gene expression of BDNF elevated in Dry-AMD patients. However, in this investigation, BDNF levels were found to be lowered in Wet-AMD patients in contrast to Dry-AMD patients. Engrossingly, a recent study assessed the BDNF levels in serum and aqueous humor using ELISA kits, and it was reported that BDNF levels were lowered in both Wet- and Dry-AMD patients compared to healthy individuals [36]. As a matter of fact, several conditions including stress, insomnia, fasting or caloric restriction, exercise, dietary supplements, and drugs were shown to alter serum BDNF levels [36, 38–40]. At this point, unlike serum analysis, one of the crucial parts of present investigation was to reveal different inclination of BDNF gene expression in PBMCs of Wet-AMD and Dry-AMD patients when compared with healthy controls.

On the other hand, CRP, C-reactive protein, is accepted as a nonspecific serum biomarker, which is mostly synthesized in the liver and adipocytes. High levels of CRP are considered a risk for heart conditions, type II diabetes, and AMD [41]. High levels of CRP were also thought to be associated with the cell and tissue damage via entailing to uncontrolled complement activation. And, CRP was determined in relatively higher levels in RPE, Bruch's membrane, choriocapillaris, and choroidal stroma in Wet-AMD eyes using alkaline phosphatase immunohistochemistry assay [41]. In supporting to this immunohistochemical finding, our results of real-time analysis revealed that the CRP expressions were significantly in patients with both types of AMD. Besides, the observed elevation level of the CRP expression was higher in Dry-AMD patients than Wet-AMD patients.

The present results clearly revealed that the *CFH*, *CX3CR1*, *FLT1*, *HIF1A*, *IGFBP3*, *MAPK3*, *SOD1*, *STAT3*, *VGEFA*, *TIMP3*, and *SERPING1* gene expressions were significantly decreased in both AMD types. The most prominent (>5 FC) decreases were observed in the expressions of *CFH*, *CX3CR1*, *FLT1*, and *TIMP3* genes. In accordance to present findings, the complement factor H (CFH) knockout mice exhibited attenuated drusen deposition and led to thinning of Bruch's membrane. Again, insufficient CFH gene exhibited synergistic action with the increased CRP expression [41, 42].

Chemokines are signal molecules that provide the migration and adhesion stimuli for the wound or inflammation site. The association of deficiencies in CXC3 chemokine receptor 1 (*CXC3R1*) with different diseases has been executed. Although the presence of studies investigating the role of fractalkin/CXC3R1 signals in ocular tissue, its exact function is still controversial. A previous investigation proved that *CXC3R1* deficiency decreased macrophage accumulation while severe neovascularization of the cornea [43]. Again, decreased expressions of *CX3CR1* mRNA and related protein in macular area indicated that these genes could play a part in the development of AMD [44]. Interestingly, *CX3CR1* positivity was not ascertained in photoreceptors or RPE cells in healthy donor eyes. Moreover, the invalidation of this gene was suggested to have association with the development of the exaggerated neovascularization which is the main outcome of Wet-AMD via inducing congregation of subretinal microglial cells [45]. In supporting this suggestion, our results indicate that the decreased level of the *CX3CR1* expression in Wet-AMD patients was demonstrably lower than Dry-AMD patients.

The results of present real-time analysis obviously introduced that the expressions of the vascular endothelial growth factor receptor 1 (*FLT1*) and tissue inhibitor of metalloproteinases 3 (*TIMP3*) genes were decreased in patients with both AMD types distinctly. There is no available molecular genetic data for supporting our findings. But a limited promotive biochemical data was recorded. Nominately, Wet-AMD patients were determined to have lower serum FLT1 levels (sFLT1) than healthy people involved in the study [46]. The conflicting results by TIMP3 on AMD pathogenesis were reported in literature. Serum TIMP-3 levels were found to significantly elevate in AMD patients when compared to healthy controls [47]. In a previous study, it was propounded that mean serum levels of TIMP3 were not significantly altered among AMD and non-AMD cases [48]. Conversely, Wet-AMD patients had significantly lower serum TIMP-3 levels than healthy subjects [49].

Nowadays, there is no effective cure for treating AMD or preventing/slowing strategies towards AMD progression. Additionally, in despite of extensive efforts, no discriminating and reliable prognostic biomarkers could be identified. The findings of this investigation clearly asserted that (I) BDNF levels were lowered in Wet-AMD but not in Dry-AMD patients, (II) the CRP expression was elevated in Dry-AMD patients, (III) the *CX3CR1* expression in Wet-AMD patients was demonstrably lower than Dry-AMD patients, and (IV) mainly *CFH*, *CX3CR1*, *FLT1*, and *TIMP3* gene levels alongside *HIF1A*, *IGFBP3*, *MAPK3*, *SOD1*, *STAT3*, *VGEFA*, *TIMP3*, and *SERPING1* gene levels were significantly reduced in both AMD types. The determined comparative gene expression alterations have potentials to clarify a part of the mechanisms underlying AMD pathogenesis, to serve effective biomonitoring of responses to the current therapies and to exhibit promising drug development targets.

## Figures and Tables

**Table 1:** Primer pairs used in real time PCR analysis.

Gene	Forward primer	Reverse primer	Reference
*BDNF*	5-CAGGGGCATAGACAAAAG-3	5-CTTCCCCTTTTAATGGTC-3	[50]
*CFP*	5′-GGAACCACCTCAATGCAAAG-3	5′-AAGCTTCTGTTTGGCTGTCC-3′	[51]
*CRP*	5′TCGTATGCCACCAAGAGACAAGACA-3′	5′-AACACTTCGCCTTGCACTTCATACTC-3′	[52]
*CX3CR1*	5′-GGGCCTGAGCCAAGCTAGAA-3′	5′-ACAGCACCTTCCAGGGATGG-3′	[53]
*FLT1A*	5′-TCCCTTATGATGCCAGCAAGT-3′	5′-CCAAAAGCCCCTCTTCCAA-3′	[54]
*HIF1A*	5′-TTCACCTGAGCCTAATAGTCC-3′	5′-CAAGTCTAAATCTGTGTCCTG-3′	[55]
*IGFBP3*	5′-GGTGTCTGATCCCAAGTTCC-3′	5′-CGGAGGAGAAGTTCTGGGTA-3	[56]
*MAPK3*	5′-TCAAGCCTTCCAACCTC-3′	5′-GCAGCCCACAGACCAAA-3′	[57]
*SOD1*	5′-ACTGGTGGTCCATGAAAAAGC-3′	5′-AACGACTTCCAGCGTTTCCT-3′	[58]
*STAT3*	5′-ACCCAACAGCCGCCGTAG-3′	5′-CAGACTGGTTGTTTCCATTCAGAT-3′	[59]
*VEGFA*	5′-CTTGCCTTGCTGCTCTACC-3′	5′-CACACAGGATGGCTTGAAG-3′	[60]
*TIMP3*	5′-TATGACTAGTAGCCCAGTGATGCTTGTGTTG-3′	5′-TATGAAGCTTATTCAGGAAAATGGCGGCATGTG-3′	[61]
*SERPING1*	5′-ATTCTCCTACCCAGCCCACT-3	5′-GGCGTCACTGTTGTTGCTTA-3′	[62]

**Table 2:** Gene expression analysis of Dry-AMD and Wet-AMD patients. Symbol (^∗^) represents statistically significant increase or decrease in gene expression (F.C.≥2).

Disease	Increased		Decreased	
	Gene	Fold change	Gene	Fold change
Dry-AMD	*BDNF* ^∗^	3.20 ± 0.22	CFH^∗^	15.35 ± 1.07
	*CR* ^∗^	5.06 ± 0.35	*CX3CR1* ^∗^	5.27 ± 0.37
			*FLT1* ^∗^	7.50 ± 0.53
	*HIF1A*	1.90 ± 0.13
	*IGFBP3* ^∗^	3.62 ± 0.25
	*MAPK3* ^∗^	5.25 ± 0.37
	*SOD1* ^∗^	3.63 ± 0.25
	*STAT3* ^∗^	3.26 ± 0.22
	*VGEFA* ^∗^	4.50 ± 0.32
	*TIMP3* ^∗^	8.19 ± 0.57
	*SERPING1* ^∗^	3.60 ± 0.25

Wet-AMD	*CRP* ^∗^	2.20 ± 0.15	*BDNF*	1.64 ± 0.11
			*CFH* ^∗^	8.15 ± 0.57
	*CX3CR1* ^∗^	8.35 ± 0.16
	*FLT1* ^∗^	5.66 ± 0.39
	*HIF1A* ^∗^	5.80 ± 0.41
	*IGFBP3* ^∗^	2.25 ± 0.15
	*MAPK3* ^∗^	4.10 ± 0.29
	*SOD1* ^∗^	9.45 ± 0.66
	*STAT3* ^∗^	7.10 ± 0.50
	*VEGFA* ^∗^	2.50 ± 0.18
	*TIMP3* ^∗^	5.40 ± 0.38
	*SERPING1* ^∗^	5.55 ± 0.39

## Data Availability

The data are available on request from Dr. Emine Cinici. E-mail: emine.cinici@atauni.edu.tr.
